# Influence of socioeconomic status on the whole blood transcriptome in African Americans

**DOI:** 10.1371/journal.pone.0187290

**Published:** 2017-12-05

**Authors:** Amadou Gaye, Gary H. Gibbons, Charles Barry, Rakale Quarells, Sharon K. Davis

**Affiliations:** 1 National Human Genome Research Institute, National Institutes of Health, Bethesda, MD, United States of America; 2 National Heart, Lung, and Blood Institute, National Institutes of Health, Bethesda, MD, United States of America; 3 Brown University, Providence, RI, United States of America; 4 Community Health and Preventive Medicine, Cardiovascular Research Institute Morehouse School of Medicine, Atlanta, GA, United States of America; Oklahoma Medical Research Foundation, UNITED STATES

## Abstract

**Background:**

The correlation between low socioeconomic status (SES) and poor health outcome or higher risk of disease has been consistently reported by many epidemiological studies across various race/ancestry groups. However, the biological mechanisms linking low SES to disease and/or disease risk factors are not well understood and remain relatively under-studied. The analysis of the blood transcriptome is a promising window for elucidating how social and environmental factors influence the molecular networks governing health and disease. To further define the mechanistic pathways between social determinants and health, this study examined the impact of SES on the blood transcriptome in a sample of African-Americans.

**Methods:**

An integrative approach leveraging three complementary methods (Weighted Gene Co-expression Network Analysis, Random Forest and Differential Expression) was adopted to identify the most predictive and robust transcriptome pathways associated with SES. We analyzed the expression of 15079 genes (RNA-seq) from whole blood across 36 samples.

**Results:**

The results revealed a cluster of 141 co-expressed genes over-expressed in the low SES group. Three pro-inflammatory pathways (IL-8 Signaling, NF-κB Signaling and Dendritic Cell Maturation) are activated in this module and over-expressed in low SES. Random Forest analysis revealed 55 of the 141 genes that, collectively, predict SES with an area under the curve of 0.85. One third of the 141 genes are significantly over-expressed in the low SES group.

**Conclusion:**

Lower SES has consistently been linked to many social and environmental conditions acting as stressors and known to be correlated with vulnerability to chronic illnesses (e.g. asthma, diabetes) associated with a chronic inflammatory state. Our unbiased analysis of the blood transcriptome in African-Americans revealed evidence of a robust molecular signature of increased inflammation associated with low SES. The results provide a plausible link between the social factors and chronic inflammation.

## Introduction

The effect of adverse environmental conditions, including social context, on health and mortality has been substantiated by numerous studies, in both human and non-human models [1–4]. The characteristics of one’s environment are largely determined by his/her socioeconomic status (SES). The effect of SES on health outcomes has been extensively studied in classical epidemiology. African Americans have disproportionately lower SES and corresponding worse health outcomes (insert reference). However, not much is known about the mechanistic pathways that link adverse social conditions to the physiological changes observed in disease state among African Americans. Studies in human social genomics aim to elucidate the molecular mechanisms, particularly the regulation of gene expression, that explain the effects of adverse social conditions such as low socioeconomic on disease and disease susceptibility [5]. Earlier studies showed that low SES is significantly associated with heart disease and its risk factors when compared to higher SES [6]. These observations, and similar others, raise the importance of understanding how human biology is affected by SES.

Findings from human social genomics studies suggest that the observed physiological response triggered by adverse social conditions is associated with a change in the gene expression profile of a specific set of genes linked to the innate immune system developed to react against threats in human ancestral environment and referred to as the Conserved Transcriptional Response to Adversity (CTRA) [5, 7, 8]. This response to adversity has been crucial for human survival in periods of threat and in modern times, can be activated by real or perceived social threats or stressors to predispose to diseases associated with perturbed immune system such as chronic inflammation. For example, Powell and colleagues demonstrated in animal model that the response to adverse social conditions involves a pro-inflammatory state characterized by increased expression of some CTRA genes in peripheral mononuclear cells [7].

Evidences of the social regulation of gene expression currently available are mainly from experimental animal model studies and controlled clinical trials [5, 7–10]. Studies have demonstrated the relationship between low SES and impaired immune system [9, 11–13]. Although studies carried out to date have provided invaluable information about genes sensitive to social influence, they have mainly focused on the CTRA set of genes. It is reasonable to postulate that other potentially important SES-responsive genes might not have been captured in the prior experiments that defined the original subgroup of genes in the CTRA set. Moreover, it is also postulated that in addition to the original CTRA subset, there are additional pro-inflammatory pathways capable of mediating the effects of adversity on the human immune system. This is particularly plausible because the effect of social status on gene expression is known to be largely context-dependent [14].

The aim of this study is to identify expression signatures that distinguish between low and high SES African American subjects as a means of establishing a contextual understanding of the mechanistic pathways mediating health. Findings may provide important insight into the biological pathway associated with SES and health in African Americans.

## Material and methods

### Data analysis

The Minority Health Genomics and Translational Research Bio-Repository Database (MH-GRID) project is a study of African American across 8 sites in the United Sates. The study enrolled self-identified, normotensive (controls with normal kidney function) and hypertensive (cases), African Americans aged between 35 and 55 years. The exclusion criteria included secondary form of hypertension, diabetes and other chronic conditions. More details on the inclusion and exclusion criteria of the MH-GRID study are detailed in section A in S1 File. The data included in this analysis is from an MH-GRID sub-study of whole blood RNA from the Morehouse School of Medicine (MSM), in Atlanta (Georgia). All participants signed a written informed consent before their participation in the study. The study was approved by the Morehouse School of Medicine, Kaiser Permanente, Grady Health System Research Oversight Committee, and the National Institutes of Health Institutional Review Boards.

The expression data consist of the messenger RNA (mRNA) sequencing data of 36 samples. RNA extraction: total RNA extraction was carried out using MagMAX^TM^ for Stabilized Blood Tubes RNA Isolation Kit as recommended by vendor (Life Technologies, Carlsbad, CA). Library preparation: total RNA samples were converted into indexed cDNA sequencing libraries using Illumina’s TruSeq sample kits (Small RNA and Stranded Total RNA respectively). After PCR amplification, the final libraries were quantitated by qPCR (KAPA Library Quant Kit, KAPA Biosystems). Expression quantification: Read counts (expression levels) were obtained using a pipeline based on BowTie2 as alignment tool and the read count were determined using RSEM.

**Fig 1:**
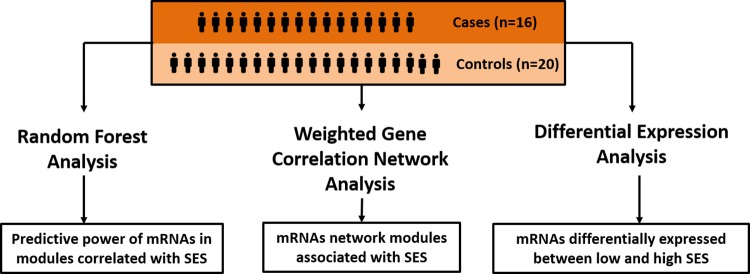
Graphical depiction of the strategy that uses 3 complementary methods.

A description of the baseline characteristics of the 36 samples (21 low SES and 16 high SES is outlined in Table 1. Education was used as a measure of SES because the literature consistently demonstrated an inverse association between cardiovascular disease mortality, morbidity, and risk factors with education [6]. Education was categorized into two groups: subjects that graduated from college/university were categorized as high SES and those who had a high school education or lower were categorized as low SES. All three analyses shown in Fig 1 were adjusted for age, gender, BMI, smoking and hypertension status by matching for those variables. For the matching BMI was categorized into 4 groups: underweight (< 18.5), normal (≥ 18.5 and < 25), overweight (≥ 25 and < 30) and obese (≥ 30). Since MH-GRID is primarily a study of hypertension, it is important to adjust for that condition. We used the R library *matchIt* [15] to match age, gender, BMI, smoking and hypertension status. For the matching parameter, we chose (exact matching) *matchIt* generates subclasses where all units (cases and controls) have the same values for the variables to match for.

**Table 1:** Baseline characteristics of the samples included in the analysis.

Characteristics	Low SES (n = 20)	High SES (n = 16)
Age (years)	45.5±5.71	46.19±5.01
BMI (kg/m^2^)	33.59±9.12	31.29±5.87
Gender (female/male)	11/9	8/8
Smoking (non-smoker/current smoker)	11/9	10/6
Hypertension (yes/no)	12/8	7/9

### Methods

An integrative approach leveraging 3 complementary methods was used to identify the most predictive and robust transcriptome pathways associated with SES. In Fig 1 the series of analyses conducted and the methods used are shown graphically. The 36 samples described in the section above were used for each of the 3 analyses in Fig 1.

#### Weighted gene co-expression network analysis (WGCNA)

WGCNA enables the investigation of the interplay (co-expression) between mRNAs and identify mRNA network modules (clusters of genes whose expression is highly correlated) and their potential relationship with the outcome of interest, SES. This analysis was conducted following the WGCNA methodology [16], in the R environment [17], in 3 steps described in ample details elsewhere [16]. A summary of those 3 steps is available from section D in S1 File. The 36 samples included in the analysis represent a sample size larger than the minimum size (15 samples) recommended by the authors of WGCNA [18].

Gene Ontology (GO) enrichment analysis was subsequently performed in R by running one-sided hypergeometric tests equivalent to Fisher's exact test [19] to identify GO terms overrepresented in network modules significantly correlated with SES. The universe (pool of genes the module-set is assessed against) consists of *Entrez Gene IDs* associated with any gene ontology term. GO terms enriched with a false discovery rate (FDR) adjusted p-value ≤ 0.05 are reported. Pathway analysis was conducted in QIAGEN’s Ingenuity® Pathway Analysis (IPA®, QIAGEN Redwood City, www.qiagen.com/ingenuity) to identify, biological pathways, disease or biological functions and upstream regulators enriched and activated in our dataset.

#### Predictive modeling using Random Forest (RF)

Random Forest (RF) like other ensemble machine learning techniques makes no assumptions about the relationship (e.g. linear) between the predictors and the outcome and can capture interactions not easily fit in regression models. It is hence a suitable method to investigate complex data like gene expression profiles and, in our analysis, estimate how well genes co-expressed in the relevant network modules can, collectively, predict SES. If a network module is truly associated with SES then one would reasonably expect the genes or a subset of the genes in that module to be good predictors of SES. An R implementation of the algorithm developed by Breiman and Cutler [20] was used for the RF analysis. More details about RF and the parameter settings used in this analysis are available from section E in S1 File.

#### Differential expression (DE) analysis

**Fig 2:**
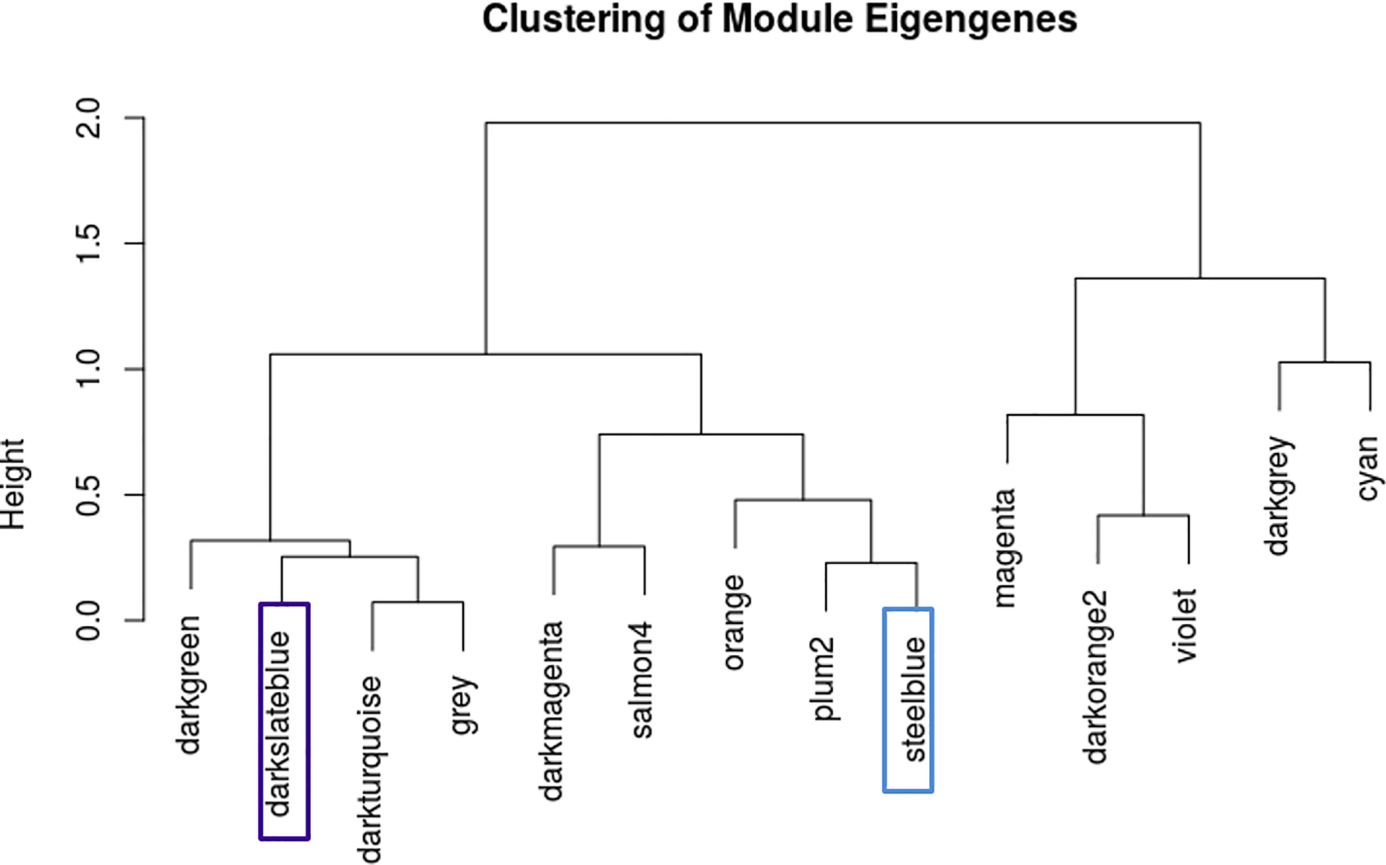
Statistical power and false discovery rate (FDR) for several expression strata.

DE analysis was carried out to identify genes with a significant difference in expression between cases (low SES) and controls (high SES) and their overlap with genes in network modules associated with SES. The R library edgeR [21] was used to examine differential expression. EdgeR fits a negative binomial model to transcripts read counts (i.e. expression) and computes likelihood ratio tests for the coefficients in the model. Transcripts with a log2 fold change (logFC) not equal to 0 and a false discovery rate (FDR) adjusted p-value ≤ 0.1 were reported as significantly differentially expressed. The relaxed FDR cut-off (0.1), compared to the conventional 0.05 is motivated by the fact that this is not a standalone analysis where we rely only on the DE results (log fold change and p-value); rather, it is considered in conjunction with the other 2 approaches to mitigate false positives. The DE analysis was adjusted for hypertension status by including it as covariate in the fitted negative binomial model. Power analysis conducted using the R library PROPER [22], showed that, with 16 replicates (16 high and 16 low SES samples), statistical power ≥ 0.8 and ≤ FDR 0.1 is achieved for genes with an average expression ≥ 10 read counts (Fig 2). Details of the power analysis are available from section F in S1 File.

#### Quality controls

For each of the 3 analyses the mRNA expression data was normalized, using the weighted trimmed mean of M-values (TMM) method, an optimal method for the normalization of mRNA count data [23]. Transcripts with an expression < 1 Count Per Million (CPM = count/sum [counts] x 1million) in at least 3 samples were excluded because results from genes with extremely low expression are not reliable; see section C in S1 File. For the DE analysis, a deviance of goodness of fit test was carried out to identify genes where the model fit was poor indicating that the dispersion estimate was away from the common dispersion, for a gene; those dispersion outliers should be carefully inspected if differentially expressed because outlying dispersion might indicate low quality or marked expression difference. Principal Component Analysis (PCA) of the mRNA expression data was carried to identify sample outliers.

## Results

A total of 15079 genes in the initial list of 27939 passed QC filters and were included in the subsequent analyses. Based on the PCA results no sample was excluded and all 36 samples were therefore included.

### WGCNA and gene ontology enrichment analyses

#### Identification of network modules

The standard protocol mentioned in the methods section and recommended by Langfelder and Horvath [16] was followed to determine the appropriate soft-thresholding power and generate the adjacency matrix (matrix of expression similarities between genes). After clustering, 59 network modules (clusters) were identified and modules whose expression profiles are very similar (dendogram cut-height ≥ 0.8) were subsequently merged. A dendogram plot of the 59 modules and the merging threshold is available in section D in S1 File. After merging modules with close expression profiles, 14 modules (Fig 2) remained.

#### Relationship between modules and SES

**Fig 3:**
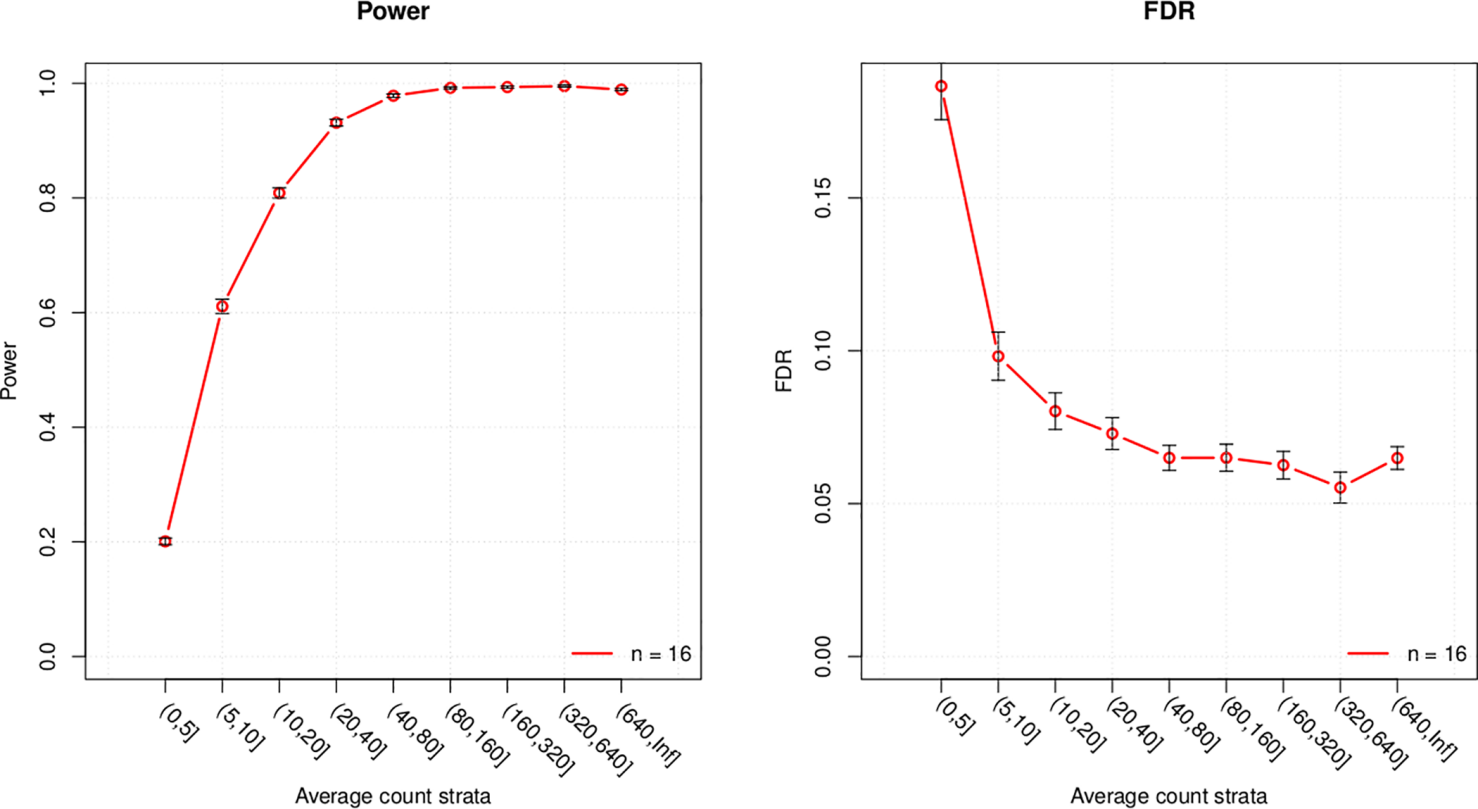
Dendogram view of the 14 network modules identified; 2 modules associated with SES are highlighted.

The correlation between SES and each of the 14 modules in the network depicted in Fig 3 was computed and a p-value, adjusted for the number of modules tested, was calculated. The statistical significance of the relationship between module and SES was assessed in two steps: first by considering the p-values of the correlation and then by considering the correlation between Module Membership (MM) and Gene Significance (GS). MM is the correlation between the gene expression profile and the module eigengene (aggregate of the expression of all genes in a module) and GS is the absolute value of the correlation between a gene and the outcome, SES. The correlation between a module and SES is in fact an association between the module eigengene and SES; hence in a plausible relationship, hub genes (genes with higher MM) would tend to be more correlated with SES leading to a positive correlation between MM and GS. Based on those two criteria, 2 modules, darkturquoise (174 co-expressed genes) and steelblue (141 co-expressed genes), were plausibly associated with SES as reported in Table 2.

**Table 2:** Correlation between two network modules and SES.

Module	Correlation	P-Value	FDR P-Value	MM-GS correlation (P-Value)
darkturquoise	0.29	0.01	0.05	0.38 (2.30E-007)
steelblue	0.44	0.004	0.003	0.38 (0.033)

#### GO terms enriched in modules associated with SES and pathway analysis

A total of 212 Gene Ontology (GO) terms were significantly enriched (FDR p-value ≤ 0.05) in the steelblue module; the top biological processes relate to stress response and immune response. No GO term was significantly enriched in the darkturquoise module, after adjusting for FDR. The full list of enriched terms in the steelblue module is available in S1 Table.

Pathway analysis conducted for the steelblue module identified:

3 pathways significantly enriched in the module and with strong evidence of activation (z-score of activation > 2): IL-8 Signaling (p-value = 3.19×10^−4^, z-score = 2.45), NF-κB Signaling (p-value = 1.20×10^−3^, z-score = 2.45) and Dendritic Cell Maturation (p-value = 1.58×10^−3^, z-score = 2.80).

1 biological process, Inflammatory Response, as the top process influenced by the co-expressed genes; the p-values of the functions range from 4.23×10^−4^ to 3.47×10^−10^.

1 upstream regulator, *CSF2*, enriched in the module and with strong evidence of activation (p-value = 1.58×10^−6^, z-score = 3.08); 14 of the 51 genes known to be regulated by CSF2 are in the steelblue module as shown in Figure D in S1 File. CSF2 was not statistically significantly differentially expressed (p-value > 0.05); however, the plot of the expression of CSF2 by SES (Figure D in S1 File) indicates a higher expression in low SES.

### Random Forest analysis

We conducted RF analyses using genes in the darkturquoise and steelblue modules as predictors and the R library VSURF to determine the set of genes that predict SES with the highest accuracy. The VSURF algorithm allows for us to carry out variable selection in 3 stages described in section E in S1 File.

The RF results, show that a subset of 55 genes from the steelblue module can collectively predict SES with an area under the curve (AUC) = 0.85 (sensitivity = 0.90, specificity = 0.69). For the darkturquoise module, a subset of 52 genes, collectively, predict SES with an AUC = 0.77 (sensitivity = 0.70, specificity = 0.69).

### Differential expression analysis

A total of 503 of the 15079 genes that passed QC are significantly differentially expressed; 405 overexpressed and 50 under-expressed in the low SES. Respectively 48 and 4 (all over-expressed in low SES) are from the steelblue and darkturquoise modules. Most importantly, all 52 genes differentially expressed, in the two network modules associated with SES, have a level of expression in the rage (strata) where nearly 100% power can be achieved (Fig 1); see detailed list in Table A in S1 File and a subset in Table 3, below.

**Table 3:** Genes significantly differentially expressed in the steelblue by pathways and upstream regulators identified in the network module.

Pathway / Upstream regulator	Gene Symbol	Gene Name	Expression Strata	Module Membership	logFC	P-Value (FDR)
IL-8 Signaling	CXCR1	C-X-C motif chemokine receptor 1	(640, Inf]	0.87	0.61	0.005
	LIMK2	LIM domain kinase 2	(640, Inf]	0.81	0.52	0.007
	CXCR2	C-X-C motif chemokine receptor 2	(640, Inf]	0.94	0.64	0.010
	GNG10	G protein subunit gamma 10	(640, Inf]	0.93	0.48	0.027
	PTGS2	prostaglandin-endoperoxide synthase 2	(640, Inf]	0.82	0.39	0.110
	PAK2	p21 (RAC1) activated kinase 2	(640, Inf]	0.85	0.17	0.617
	IQGAP1	IQ motif containing GTPase activating protein 1	(640, Inf]	0.88	0.18	0.657
NF-κB Signaling	PELI1	pellino E3 ubiquitin protein ligase 1	(640, Inf]	0.94	0.46	0.035
	IL1R1	interleukin 1 receptor type 1	(640, Inf]	0.86	0.55	0.035
	TLR1	toll like receptor 1	(640, Inf]	0.97	0.43	0.051
	TLR4	toll like receptor 4	(640, Inf]	0.93	0.43	0.078
	TLR8	toll like receptor 8	(640, Inf]	0.89	0.22	0.499
	GSK3B	glycogen synthase kinase 3 beta	(640, Inf]	0.81	0.12	0.678
Dendritic Cell Maturation	FCGR3B	Fc fragment of IgG receptor IIIa	(640, Inf]	0.89	0.76	0.011
	FCGR2A	Fc fragment of IgG receptor IIa	(640, Inf]	0.95	0.5	0.041
	TLR4	toll like receptor 4	(640, Inf]	0.93	0.43	0.078
	MAPK14	mitogen-activated protein kinase 14	(640, Inf]	0.83	0.3	0.249
	CD58	CD58 molecule	(640, Inf]	0.84	0.25	0.382
	IFNAR1	interferon alpha and beta receptor subunit 1	(640, Inf]	0.88	0.19	0.544
CSF2	GK	glycerol kinase	(640, Inf]	0.9	0.49	0.018
	IL1R1	interleukin 1 receptor type 1	(640, Inf]	0.86	0.55	0.035
	TLR1	toll like receptor 1	(640, Inf]	0.97	0.43	0.051
	CSF2RB	Colony Stimulating Factor 2 Receptor Beta	(640, Inf]	0.89	0.44	0.066
	FPR2	Formyl Peptide Receptor 2	(640, Inf]	0.85	0.4	0.074
	TLR4	toll like receptor 4	(640, Inf]	0.93	0.43	0.078
	CLEC7A	C-type lectin domain family 7 member A	(640, Inf]	0.88	0.38	0.093
	PTGS2	prostaglandin-endoperoxide synthase 2	(640, Inf]	0.82	0.39	0.110
	SOD2	Superoxide Dismutase 2, Mitochondrial	(640, Inf]	0.96	0.4	0.213
	RRM2B	Ribonucleotide Reductase Regulatory TP53 Inducible Subunit M2B	(640, Inf]	0.9	0.25	0.329
	LAMP2	Lysosomal Associated Membrane Protein 2	(640, Inf]	0.95	0.25	0.388
	BID	BH3 Interacting Domain Death Agonist	(640, Inf]	0.84	0.19	0.508
	MCL1	BCL2 Family Apoptosis Regulator	(640, Inf]	0.93	0.28	0.512
	MDM2	MDM2 Proto-Oncogene	(640, Inf]	0.84	0.11	0.739

The DE genes were inspected for sample outliers to ensure DE is not driven by outliers; all 50 genes from the 2 modules passed that check as illustrated by the plots of 12 genes from the steelblue module in Figure H in S1 File. The Full DE results for the steelblue and darkturquoise module are reported respectively in S2 and S3 Tables.

### CTRA genes

The expression levels of 35 CTRA genes are available from the MH-GRID data; 28 genes passed QC filtering and were included in the analyses. One of the 28 genes, PTGS2, is in the steelblue module.

An RF analysis was run with the 28 CTRA genes that passed QC filters and the 36 samples; the results of that analysis indicate that, collectively, the CTRA set is a poor predictor of SES (AUC = 0.52). However, the top 5 genes (FOSL2, IL1B, PTGS1, PTGS2 and IFI44) by predictive power collectively predict SES with AUC = 0.77.

In the DE analysis of the 15079 genes that passed QC, 9 CTRA genes are differentially expressed but 7 of those were flagged as dispersion outliers based on deviance of goodness of fit test. A close look at the expression of those genes with and without sample outliers reveals that the difference between high and low SES is markedly influenced by 2 expression outliers in one group (Figure I in S1 File). The genes FOSL2, IFI16, IL1B and PTGS2, the latter 2 borderline significant, are overexpressed in low SES; the implication of this observation is discussed in the next section.

## Discussions

### Summary of findings

Weighted gene co-expression network analysis (WGCNA) of 15079 genes, in a matched low/high SES sample set to mitigate the influence of potential confounders, and identified 14 network modules (clusters of co-expressed genes). Two modules named ‘darkturquoise’ and ‘steelblue’, with respectively 174 and 141 co-expressed genes, were significantly and positively correlated with SES; these 2 modules are over-expressed in low SES. The top GO terms significantly enriched in the steelblue module relate to stress response and immune response. Pathway analysis revealed 3 pathways (IL-8 Signaling, NF-κB Signaling and Dendritic Cell Maturation); one biological function (Inflammatory Response); and one upstream regulator gene (CSF2) significantly enriched and activated in the steelblue module.

Random Forest (RF) analysis identified a group of 55 genes, in the steelblue module, that collectively predict SES with high accuracy; 12 of them including CXCR1, CXCR2, PTGS2, TLR1 are involved in the pathways found activated in the data and over-expressed in the low SES group. These RF results represent a confirmation, through machine learning, of the relationship between the steelblue network module and SES.

Finally, the expression of the genes in the modules was contrasted between the 16 low and 20 high SES subjects. A total of 48 genes in steelblue module were found to be significantly differentially expressed between the two groups; 21 of those are among the subset of 51 genes that predicts SES with high accuracy (AUC = 0.85).

As previously mentioned, the strategy of investigating the data through three methods was motivated by the aim to achieve the most robust results. The results for the steelblue module seem to be the most reliable based on the overlap between the results of the 3 analyses. Therefore, the remainder of this section focuses on the steelblue module of co-expressed genes which we refer to as the module.

### Low SES is associated with pro-inflammatory state group

IL-8 (Interleukin-8) is a chemokine produced in various cells such as macrophages and endothelial cells which play an important role in inflammation, angiogenesis and tumor growth. IL-8 is pro-inflammatory and is known to recruit and activate neutrophils at inflammation sites [24]. Seven of the genes in the module (CXCR1, CXCR2, GNG10, IQGAP1, LIMK2, PAK2 and PTGS2) are involved in IL-8 Signaling pathway. CXCR1 (IL-8 receptor type 1) and CXCR2 (IL-8 receptor type 2) are cell surface receptors of IL-8 expressed in neutrophils, monocytes and endothelial cells. CXCR1, CXCR2, GNG10 and LIMK2 are significantly over-expressed in the low SES group. CXCR1, CXCR2 and LIMK2 are among the subset of 55 genes that predict SES with a high accuracy. All 7 genes involved in IL-8 Signaling, are over-expressed in low SES. Although the FDR adjusted p-value of the other 3 genes did not reach statistical significance, their combined effect and the fact that IL-8 signaling was activated in the data provides evidence of a pro-inflammatory state.

Nuclear factor-κB (NF-κB) are transcription factors that regulate the expression of genes involved in biological processes such as innate and adaptive immunity, inflammation and stress responses [12]. Six genes (GSK3B, IL1R1, PELI1, TLR1, TLR4 and TLR8) over-expressed in low SES and present in the module are involved in the NF-κB Signaling pathway activated in the expression data. The role of Toll-like receptors (TLRs), in pathogen recognition and activation of innate immune response is well established. TLRs signaling through the Myeloid differentiation primary response gene 88 (MyD88) induces the activation of mitogen-activated protein kinases such as MAPK14 (over-expressed in low SES) implicated in various biological processes including inflammatory response. Interleukin 1 Receptor type 1 (IL1R1) mediates cytokine-induced immune and inflammatory responses; the protein it encodes acts as a receptor for IL-1α, IL-1β responsible of the activation of the immune response system and IL-1RA which inhibits the pro-inflammatory effect of IL-1α and IL-1β.

The third pathway enriched in the module is Dendritic Cell Maturation. Dendritic cells (DC) are antigen-presenting cells (APCs) which play a key role in the regulation of the adaptive immune response [25]. Immature DCs capture antigens, process them, and present them on the cell surface. Maturation of DCs can be triggered by pathogens, cytokines and other cells of the immune system. DCs detect pathogens through pattern recognition receptors (PRRs) such as TLRs. Activated TLRs in turn trigger MAPK pathways which leads to the activation of transcription factors such as NF-κB whose evidence of activation and role in immune response we already described. Six of the genes in the module (CD58, FCGR2A, FCGR3B, IFNAR1, MAPK14 and TLR4) are involved in Dendritic Cell Maturation pathway; these 6 genes are all over-expressed in low SES.

Colony-stimulating factor 2 (CSF2) was identified as an activated upstream regulator of 14 genes in the module (Figure D, in S1 File). CSF2 is a pro-inflammatory cytokine implicated in many diseases, including diabetes, rheumatoid arthritis and cancer, through its links to other pro-inflammatory cytokine such as tumor necrosis factor (TNF) and IL-1 [26]. A recent study by Wand et al. provided evidence that CSF2 can be targeted by an anti-inflammatory compound in the treatment of rheumatoid arthritis [27].

Further expression pattern pointing to inflammation relates to TET2 one of the top two hub genes (the other is TRL1) in the steelblue module; these 2 genes have both a Module Membership (MM) value of 0.97, MM is a measure of how well a gene belongs to a module (cluster). The action of TET genes such as TET2 in regulating chromatin architecture and gene transcription and modulating DNA methylation was known [28–30] but it was only recently that the role of TET2 in the resolution of inflammation through the repression of IL-6 has been uncovered [31]. Two decades back Tosato and Jones already showed that IL-1 induces the expression of IL-6 in peripheral blood monocytes [32]; our results show evidence of over-expression, in low SES, of IL-1 receptor 1 (IL1R1) which suggest a higher expression of IL-6 in the same group.

Although the set of 28 CTRA genes did not predict SES well, a subset of 5 genes provided a reasonably good prediction. Four of those 5 genes, FOSL2, IL1B, PTGS1, PTGS2 are pro-inflammatory genes, previously reported as up-regulated under adverse social conditions [5, 33] and one of them, PTGS2, is in the module involved in the pathways discussed above. Two of these genes FOSL2 and PTGS2 as well as other CTRA genes such as FOS and IL8 are differentially expressed with a raw p-value ≤ 0.05. One could argue that the CTRA genes should not be penalized by FDR correction since CTRA are a priori candidates for SES. Therefore, the pro-inflammatory CTRA signal detected in this study confirms what has been reported previously about CTRA. The less stronger CTRA pro-inflammatory genes signal observed in our study, and the opposite direction of the fold change of IFI16, IFI27 and IRF2 (genes involved in type 1 IFN reported as down-regulation under adverse conditions but up-regulated in low SES in our analysis) might be due to different context; transcriptomic expression is known to be highly context-dependent [14] and the social environment of African Americans and the psycho-social factors affecting them, particularly those at the lower end of the socioeconomic spectrum, are quite different from that of the earlier studies which consisted predominantly of analyses of white populations.

Inflammation is a natural process in immune response but its excessive or persistent effect (chronic inflammation) can lead to vascular dysfunction a mechanism by which inflammation can for example promote vascular disorders such as hypertension, atherosclerosis and stroke [34–37] which are all disproportionate in African Americans.

### SES links to transcriptome

Previous studies have identified links between low SES and dysregulation of immune processes related to disease. Chen et al. showed an association between low SES and increased expression in certain immune response genes, including IL-5 and INFγ, in adolescents with asthma [9]. In another work on asthma Chen and Cole reported an over-expression of genes regulating inflammatory processes in children from low SES background [11]. Dowd and Aiello measured cytomegalovirus (CMV) antibody levels as indirect marker of cell-mediated immunity and found it associated with SES measured as level of education in a sample of non-Hispanic white (86% of the sample set), non-Hispanic black and Mexican Americans from the National Health and Nutrition Examination Survey (NHANES) III; low SES subjects had higher levels of CMV antibodies [38]. The results of these studies support the existence of socioeconomic differentials in immune response, one of the top gene ontology enriched in our analysis.

Lower SES has been consistently and reliably linked to many social (e.g. crime, crowding, discrimination) and environmental (e.g. pollution, hazards) conditions that act as stressors. Persistent stress referred to as chronic stress is associated with poorer health outcomes. Several studies have confirmed the relationship between stress and vulnerability to infectious disease, extent and intensity of inflammatory response, recovery from illness, reactivation of latent viruses, and heart and kidney disease [34, 35, 39–44]. Our analysis seems to substantiate these earlier findings. Cellular response to stress is the top over-represented ontology in the expression data. The transcriptomic disturbances we observed on the low SES group can stem from any of the aforementioned psychosocial stressors that can adversely affect behaviors including but not limited to dietary and sleep behaviors which in turn have consequences on physiologic systems governing the higher prevalence of chronic inflammation and metabolic disturbances associated with poor health outcomes in low SES individuals.

### Strengths, limitations and further work

We used a carefully thought design, stringent QC and different methods and techniques (WGCNA, RF and DE) to reduce false positive signals. This is among the first analysis to leverage the robustness of these three approaches to deal with the complexity of RNA-seq data. Although our approach increases certainty about the results it might leave out genes that would have been identified with less stringent criteria.

The analysis of whole blood provides a bigger picture because signals from many tissues are captured. However, information from certain tissues cannot be captured or can only be partially captured from whole blood. Nevertheless, it is reasonable to consider whole blood as a good source for the investigation of complex/chronic conditions which most likely involves multiple tissues, pathways and cell types.

The analyses were conducted with a modest sample size because we wanted to contrast the extremes of education (college degree and further versus high school or less) to capture a stronger signal. We did not include individuals with some college education in the high SES group because preliminary analysis, not reported here, showed an increased noise in the expression data that affected the power to detect difference between the two groups.

Follow-up analyses are planned using microRNA data to identify microRNAs regulating the genes reported in this analysis and using methylation data to have a more comprehensive picture that might uncover an epigenetic signature of low socio-economic status, at the cellular level.

### Conclusions

The relationship between low SES and disease have long been known. In their work untitled ‘The Health Needs of Disadvantaged Client Groups’ Illsley and Mullen reported the association between low SES and each of the 14 major cause-of-death categories in the International Classification of Diseases [45]. However, the search for molecular mechanisms that can shed light on the link between low SES and disease or disease risks has started only very recently. We report biological pathways that link low SES to perturbed immune function and increased inflammation.

A dysregulated immune system can have multiple consequences including but not limited to: impaired ability to identify host cells leading to auto-immune disease where the body attacks its own cells, inability to efficiently fight pathogens that enter the body, and potentially keeping the body in heightened state of ‘danger/stress alert’ (inflammation and oxidative stress) which when prolonged leads to many chronic diseases. A recent study showed that chronic inflammation accounted for 30% of the association between indicators of low socioeconomic status across the life course and an increased risk of later diabetes [46].

To our knowledge this the first study of this kind in African Americans, a population that is understudied despite the fact that it is among the groups that experience the most adverse effect of low SES and its correlates such as such as poverty, poor health and lower education [47–49].

## Supporting information

S1 File Further description of data and methods. (DOCX)

S1 Table Gene ontology enrichment analysis results. (XLSX)

S2 Table Differential expression analysis results and genes’ module membership values for the steelblue network module. (XLSX)

S3 Table Differential expression analysis results and genes’ module membership values for the darkturquoise network module. (XLSX)

S4 Table Expression and phenotype data used for the analysis. (XLSX)
