# Supplementary material for: Influence of socioeconomic status on the whole blood transcriptome in African Americans
Source: PLoS One. 2017 Dec 5;12(12):e0187290. doi: 10.1371/journal.pone.0187290 (PMC5716587; doi:10.1371/journal.pone.0187290)
Supplement: S1 File — (DOCX) [file pone.0187290.s001.docx]

Supplementary Material S1

1. **MH-GRID inclusion and exclusion criteria**

**Inclusion Criteria**

- - Self-identified African-Americans males or females ages 30-55 years.

**Cases:**

- - Severe-Controlled Hypertension (SCH): SBP ≤ 140 and/or DBP ≤ 90 mmHg on a stable regimen (≥6 months) with ≥ 2 anti-hypertensive drugs (must include a diuretic).
  - Severe-Resistant Hypertension (SRH): SBP > 140 and/or DBP > 90 mmHg on a stable regimen (≥3 months) of ≥ 3 drugs (must include a diuretic).

**Controls:** Individuals with optimal blood pressure: ≤ 120/80 mmHg and normal kidney function (eGFR > 90 ml/min).

**Exclusion Criteria**

- Failure to meet the inclusion criteria.
- Secondary forms of hypertension.
- Primary chronic kidney disease or proteinuria unrelated to hypertension.
- Chronic diseases that may secondarily compromise renal function such as diabetes, chronic congestive heart failure, HIV or liver disease.
- Patients with recent hospitalizations (< 3 months).
- Unable to give informed consent.
- Pregnant or lactating women.

1. **RNA extraction and quantification**

**mRNA Isolation**

Total RNA extraction was carried out using the MagMAX™ for Stabilized Blood Tubes RNA Isolation Kit (Life Technologies, Carlsbad, CA) following manufacturer’s recommendation. Briefly, Tempus-stabilized blood (~9 mL) was diluted with PBS and centrifuged at 5000 × g for 15 minutes at 4 °C to pellet the crude RNA. The RNA pellet was digested with Tempus Proteinase and Turbo DNase, and captured using RNA Binding Beads and a magnetic stand. The beads were washed with wash buffers and RNA eluted in 20 uL Elution Buffer. To assess RNA yield and quality, a NanoDrop Spectrophotometer (Thermo Scientific, Wilmington, DE) was used to measure RNA concentration in 260/230 ratios. Additionally, an aliquot of the RNA was analyzed on an Agilent 2200 TapeStation (Agilent Technologies, Santa Clara, CA) for RINe calculation using the RNA ScreenTapes.

**mRNA library preparation**

The data used in this analysis consist of whole transcriptome mRNA sequencing data of 86 samples from samples collected at MSM as part of the MH-GRID study. The mRNA was converted into cDNA libraries using the Illumina TruSeq Stranded Total RNA sample preparation kit (Illumina # RS-122-2303). Total RNA samples concentrations were normalized, and ribosomal RNA (rRNA) removed using biotinylated probes that selectively bind rRNA species. The resulting rRNA-depleted mRNA was fragmented using heat in the presence of divalent cations, with fragmentation times varying based on input mRNA degradation. Fragmented mRNA was converted into double-stranded cDNA, with dUTP utilized in place of dTTP in the second strand master mix. The resulting molecules were amplified via polymerase chain reaction (PCR). Final libraries were quantified by qPCR (KAPA Library Quant Kit, KAPA Biosystems # KK4824), normalized and pooled. Pooled libraries were bound to the surface of a flow cell and each bound template molecule was clonally amplified up to 1000-fold to create individual clusters. Four fluorescently labelled nucleotides were then flowed over the surface of the flow cell and incorporated into each nucleic acid chain. Fluorescence was measured for each cluster during each cycle to identify the base that was added to each cluster.

**mRNA expression quantification**

The quantification of mRNA gene-level and isoform-level expression values was done in 3 steps:

(1) Adapter trimming was conducting with FastqMcf to remove remnants of sequencing primers/adapters and low-quality regions from the raw RNA-Seq read data, and hence improves subsequent alignment rates.

(2) Reads were aligned to the transcriptome, using BowTie2 and the relevant reference genome (hg38).

(3) Finally, the expression level is measured using RNA-Seq by Expectation Maximization (RSEM).

1. **Quality controls**

Transcripts with an expression < 1 count per million (CPM =count/sum [counts] x 1million) in at least 3 samples were excluded because results from genes with extremely low expression are not very reliable. Read count distribution before and after filtering of low count genes in shown in Figure 1.

Figure A: Distribution of read counts before (A) and after (B) filtering out genes with less than 1 CPM.


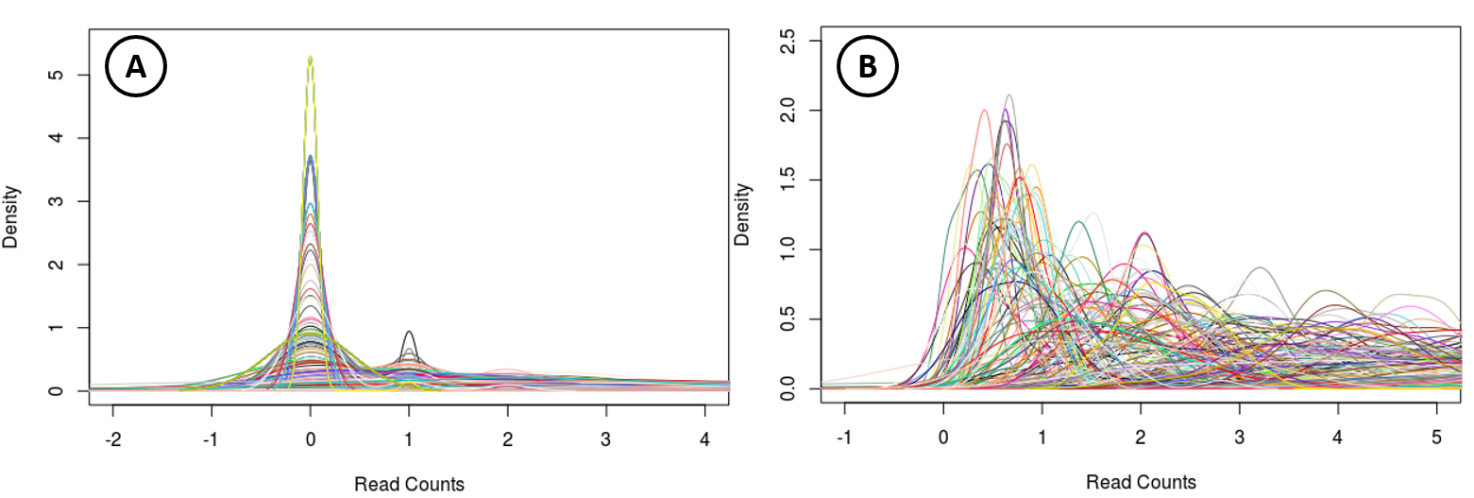


1. **WGCNA**

**WGCNA steps and our parameter settings works**

The aim of this network analysis is to investigate the interplay between genes to identify modules (clusters of genes whose expression is highly correlated) and the relationship between those modules and the phenotype of interest, SES. This analysis was conducted following the WGCNA methodology, in the R environment and in 3 steps.

1. Network construction: The network consists of all the genes that passed quality control (QC) filters. In the network, each gene is a node and closely related genes (i.e. co-expressed genes) form a module. First, a co-expression similarity matrix, s_ij_, that holds correlation values between genes is computed. Then s_ij_, is transformed into an adjacency matrix, a_ij_, a matrix that tells whether any two genes have a correlation ≥ τ, a threshold to determine if two genes are connected (close). In an un-weighted network a_ij_ takes the values 0 or 1, as shown in the mathematical expression below, and τ is called a ‘hard’ threshold.

$$\text{if }s_{\mathrm{ij}} \geq\tau\to a_{\mathrm{ij}}= 1 else a_{\mathrm{ij}}=0$$

However, such hard threshold does not reflect the continuous nature of biology. Therefore, weighted networks which allow for a_ij_ to take any values between 0 and 1 represent a more appropriate framework; τ is then called a ‘soft’ threshold. A critical decision is the choice of an appropriate τ. An adequate threshold τ is one that leads to a scale-free topology network i.e. a structured network with central (hub genes) and peripheral nodes; as opposed to a random network. A characteristic of a scale-free topology network is that the probability that a node has k connections follows a power law P(k) ~ k^-γ^; for a perfect scale-free topology network the correlation between P(k) and k is 1. In this analysis, the choice of the threshold was verified to ensure the requirement of a scale-free topology network is fulfilled: ρ_P(k),k_ ≥ 0.8.

1. Identification of network modules: After choosing an appropriate threshold, modules are identified through hierarchical clustering using the Unweighted Pair Group Method with Arithmetic Averaging (UPGMA) method ([Michener and R., 1957](#_ENREF_5)) to build a dendogram, a diagram that hierarchically nests genes into increasingly more inclusive clusters. The clustering is based on information from a topology overlap matrix (TOM), a matrix that combines co-expression information from the adjacency matrix and topological similarity ([Dewey et al., 2011](#_ENREF_2); [Yip and Horvath, 2007](#_ENREF_6)). The minimum size of the modules to detect was set to 10 to ensure small as well as large modules are detected. Subsequently modules with very similar expression (those very close in the dendogram) were merged.
2. The relationship/association between the modules identified in the previous step and SES were then investigated. The aggregated expression of each module, the first principal component of the reduced data, termed module eigengene, is computed and its correlation with SES status determined. The relationship between a module and SES is reported as significant if 2 conditions are fulfilled:
3. The false discovery rate (FDR) adjusted p.value of the correlation is ≤ 0.05.
4. The correlation between Module Membership (MM) and Gene Significance (GS) is > 0 with a p.value ≤ 0.05. MM is the correlation between the expression profile of a gene and the module eigengene (aggregated expression of all genes in a module, the 1^st^ principal component); MM takes values between 0 and 1 and tells ‘how well a gene belongs to a module’; hub genes have an MM value closer to 1. GS is the absolute value of the correlation between a gene and the outcome, SES. The correlation between a module and SES status is in fact a correlation between the module eigengene and SES status. Therefore, in a biologically plausible module-phenotype association, hub genes would be more correlated with SES than genes at the fringes of the module and this leads to a positive correlation between MM and GS.

**Scale-free network topology and clustering in our data**

A soft-threshold that ensures the network of genes has the characteristics of a scale-free network topology as shown in Figure 2 where the relationship between log10(k) and log10(P(k)) follows the power law with a correlation of 0.8.

Figure B: Plot k vs. P(k) to verify scale-free topology requirement is fulfilled.


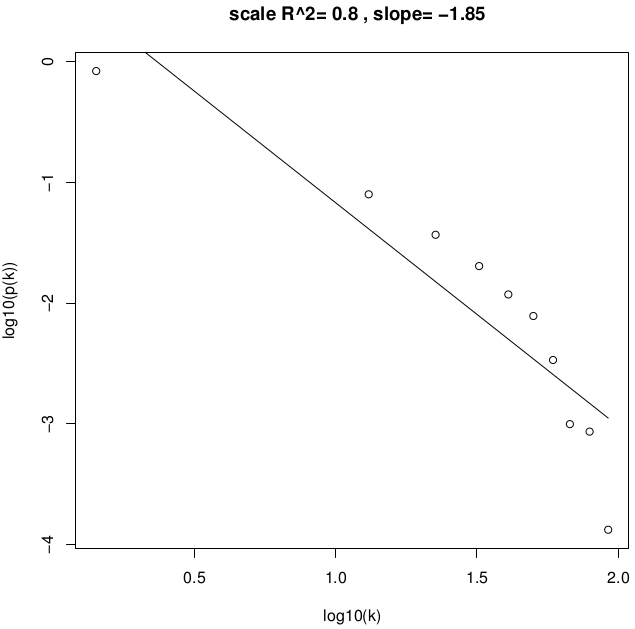


After clustering 59 modules (clusters) were identified and modules whose expression profiles are very similar (dendogram cut-height ≥ 0.8) were subsequently merged as shown in Figure 3.

Figure C: 59 modules identified after agglomerative clustering using the complete-linkage method and adaptive branch pruning. The line across the dendrogram indicates the threshold used to merge modules whose expression profiles were very similar (correlation ≥ 0.8).


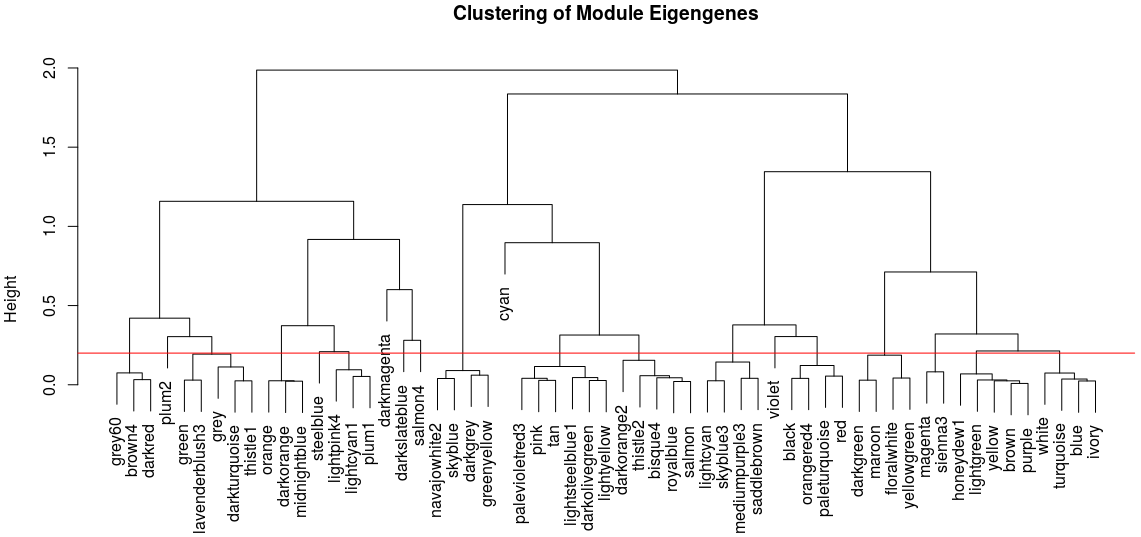


Figure D: (A) Expression of CSF2 by SES category and (B) Network view of the relationship between CSF2 and 14 downstream genes in the steelblue module.


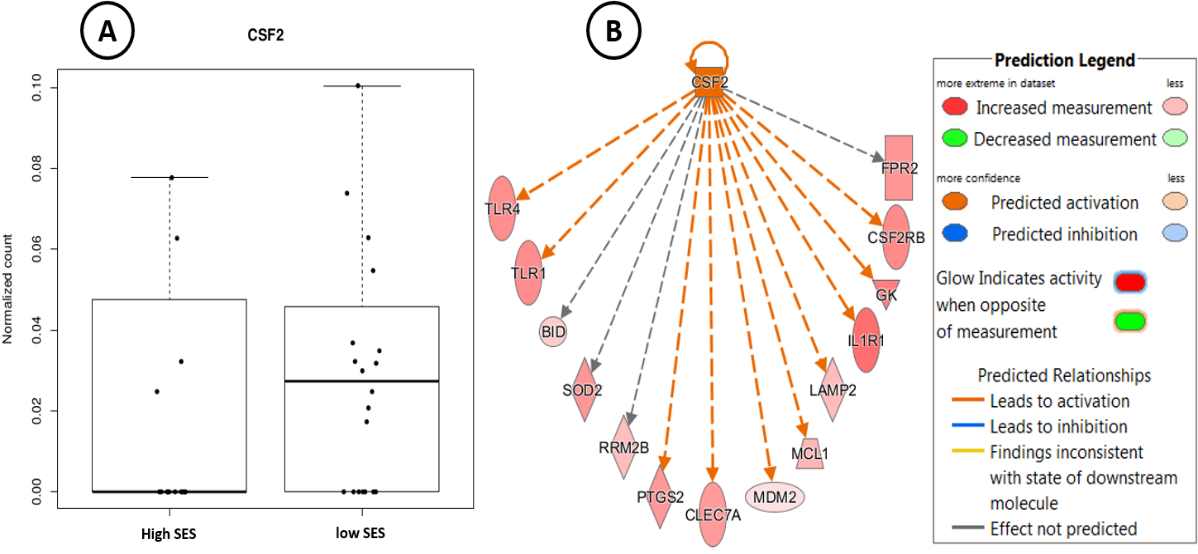


1. **Random Forest Analysis**

**Method and our parameter settings**

In RF, cross-validation is not needed and there is no distinct test set because the classification error is estimated internally ([Breiman, 2001](#_ENREF_1)): For each tree, a subset of all the samples, is drawn by sampling with replacement (bootstrap) and the rest of the data are left out. In this analysis, we generated a forest with a large number of trees (ntree), 10000, for robust prediction estimates. For each tree, the number of variables (genes) sampled (mtry) as candidates at each split is $\frac{p}{2}$ where p is the total number of genes. The samples left out represent the out-of-bag (OOB) set used to get an unbiased estimate of the misclassification error of the tree.

RF provides a Variable Importance Measure (VIM), a score that denotes the variable’s predictive power obtained as follows: for each tree, the number of correct classifications (i.e. number of votes cast for the correct class) in the OOB set is counted; then the values of the variable are permuted and the number of correct classifications in that perturbed OOB set is counted. Finally, the number of correct classifications in the un-perturbed OOB set is subtracted from that in the perturbed OOB set; the VIM of the variable is the average of the of the output of that operation across all the trees of a forest. In our RF analyses the number of permutations (*nPerm*) was set to 1000.

All the variables in the RF output ranking are not necessarily *important* i.e. true predictors; some are noise. It is therefore important the select the true predictors, a process called *variable selection*. We carried out variable selection using a method by Genuer et *al*. recently implemented in the R programming language and described elsewhere ([Genuer et al., 2010](#_ENREF_3), [2015](#_ENREF_4)). This method has the particularity of using a heuristic approach where the threshold to sift out noise predictors is derived from the data and is hence not an arbitrary cut-off not related to the data ([Genuer et al., 2015](#_ENREF_4)).

**Description of variable selection and results**

The plots in Figure 4 are for the steelblue module to illustrate the process. Results for both modules are summarized in Table 4:

1. Ranking of variables: 500 forests of 10000 trees each are generated and the variables ranked by mean VIM (Figure 4A).
2. Selection of true predictors: the standard deviations of VIM are then used to estimate a threshold value for VIM. The threshold determination is described with ample details elsewhere ([Genuer et al., 2010](#_ENREF_3), [2015](#_ENREF_4)); briefly, the threshold (dotted horizontal red line in Figure 4B) is set to the minimum prediction value given by a classification and regression tree (CART) model fitting the standard deviation curve (the minimum of the green piece-wise function in Figure 4B). A total of 55 genes with an averaged VIM > this threshold (represented by the red line in Figure 4A) are selected.
3. Model performance of the true predictors measured as OOB error rate: Figure 4C indicates that the lowest OOB error (0.17) is achieved with the first 11 and 12 ranked genes which can collectively predict SES with an AUC of 0.87. When all 55 selected genes, from the steelblue module, are included in the model the OOB error increases slightly to 0.19 and AUC = 0.85.

**Figure E: The 3 main steps of the variable selection shown graphically: (A) ranking of variables by mean VIM, (B) standard deviation of VIM for each variable and (C) OOB error for nested models fitted across the 55 true predictors, the red dashed line indicates the mean OOB error.**


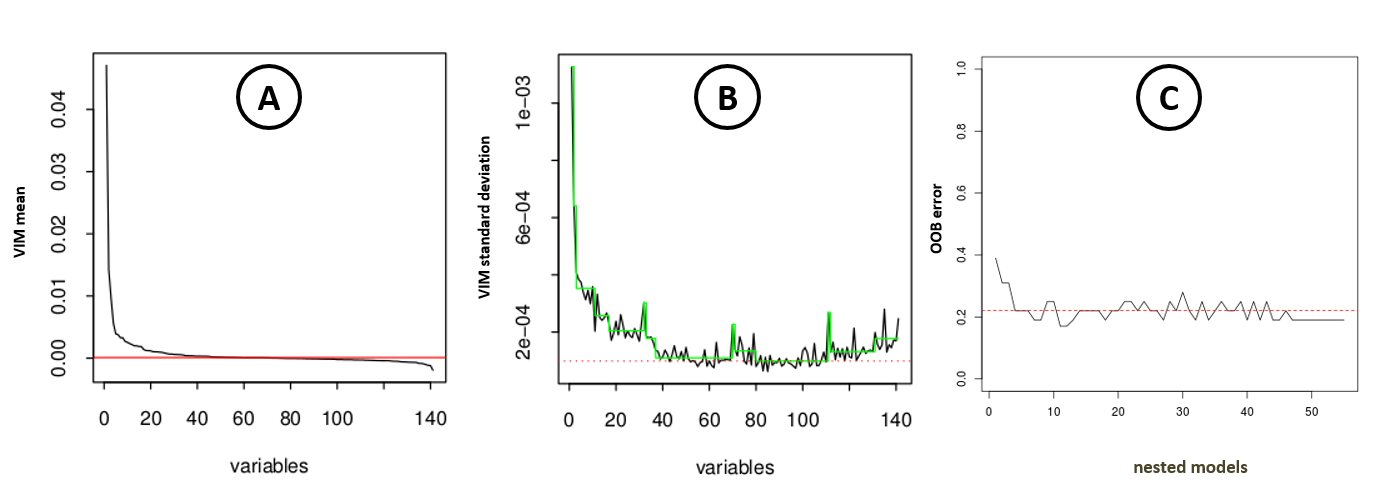


**Figure F: (A) VIM rank and (B) AUC for the 55 steelblue module genes retained after variable selection.**


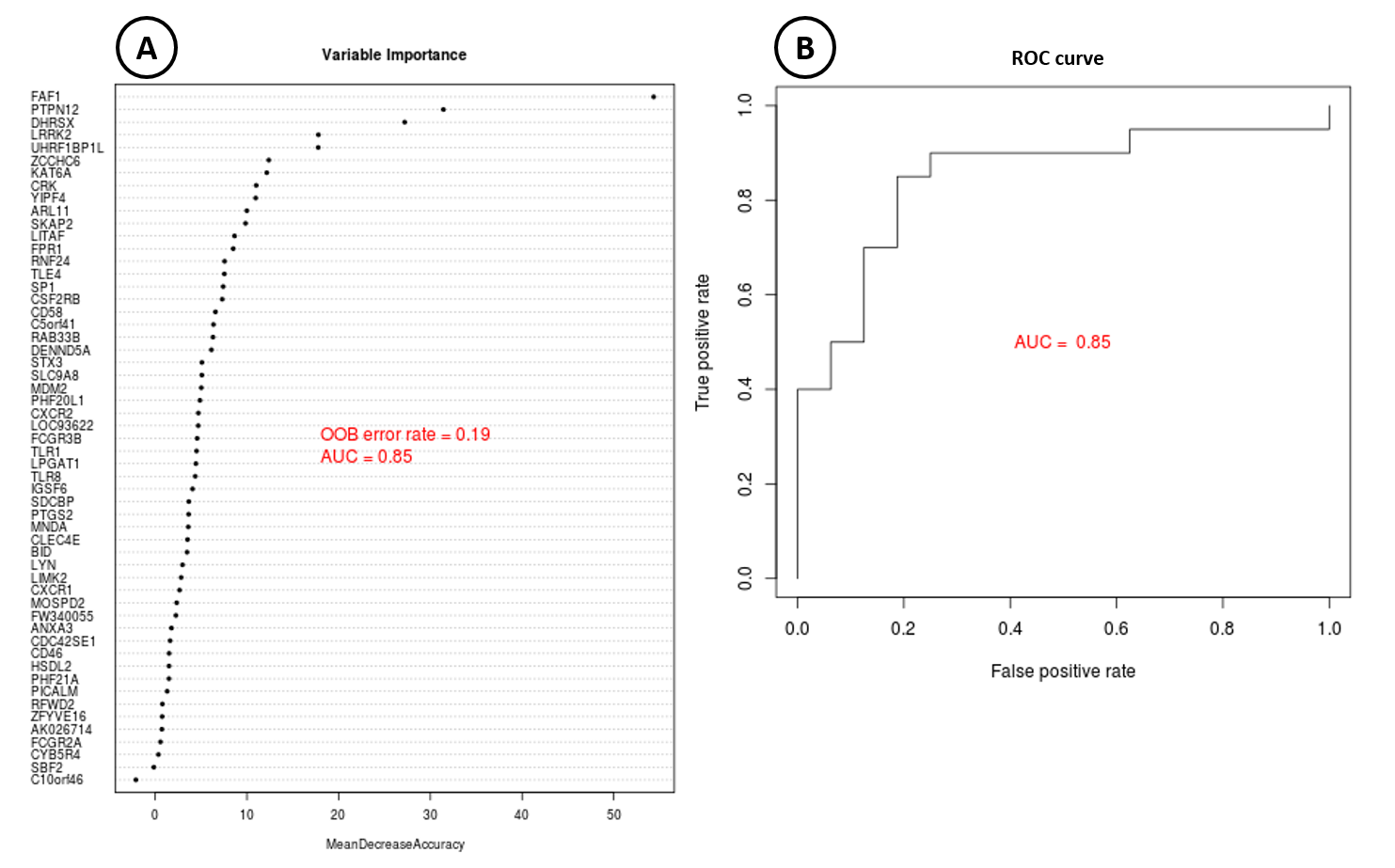


1. **Differential Expression Analysis**

**Power Analysis**

Calculations were conducted using the R library PROPER which takes into account factors that influence power such as sequencing depth, distribution of the mean expression level, and threshold for filtering out molecules and evaluates power empirically. Power was assessed by evaluating success rate over 300 simulation runs. Success rate was determined by comparing the p-values to the specified type I error rate (alpha = 0.05) to reject to null hypothesis. In the below Figure 3, the plot on right indicates that, 16 pairs (16 high and 16 low SES subjects), a statistical power ≥ 0.8 and FDR < 0.1 is achieved to identify differentially expressed gene whose average expression ≥ 10 read counts. For mRNAs with an average expression between 5 and 10 read counts the power achieved is 0.6 and FDR < 0.1.

Figure G: Average statistical power and false discovery rate (FDR) for several expression strata given the characteristics of the MH-GRID mRNA data.


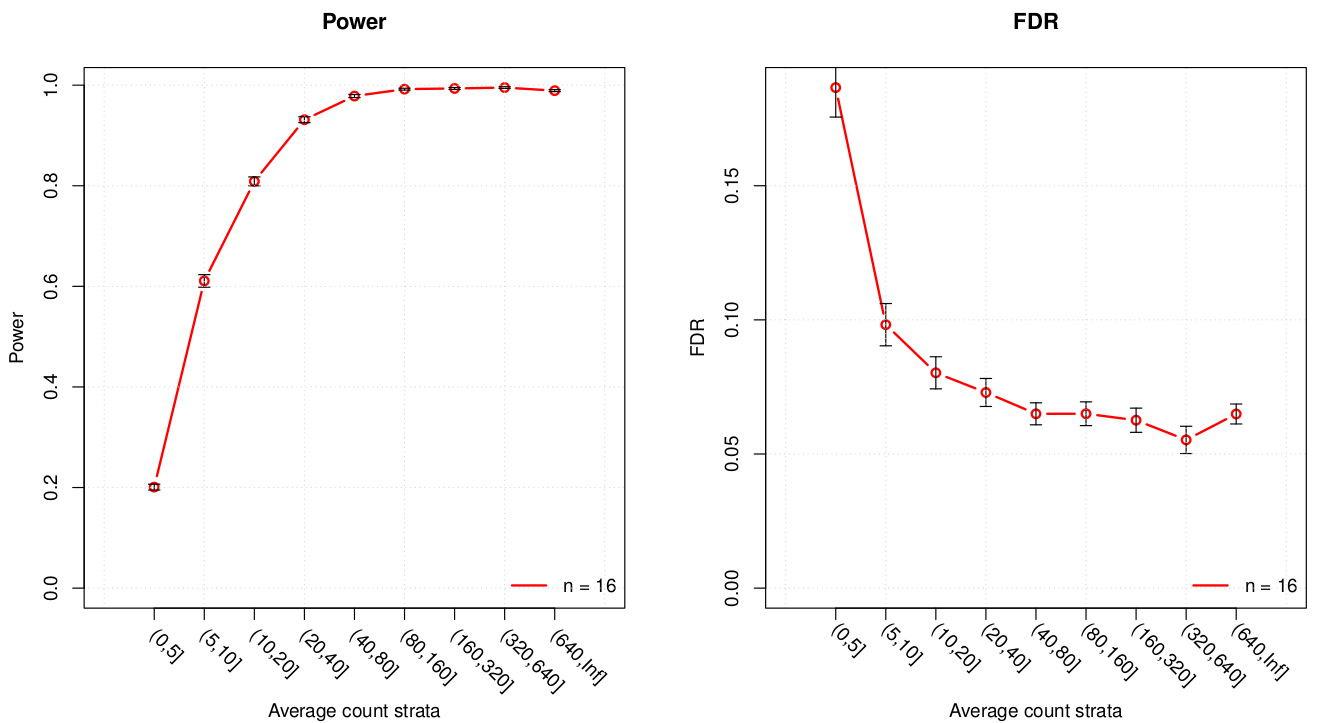


Table A: Expression levels of genes differentially expressed in the two network modules associated with SES.

| **Expression strata** | **Genes statistically significantly differentially expressed** |
| --- | --- |
| (0, 5] | - |
| (5, 10] | - |
| (10, 20] | - |
| (20, 40] | - |
| (40, 80] | - |
| (80, 160] | BC033528 |
| (160, 320] | C7orf53, TPTEP1, CBFA2T3 |
| (320, 640] | DHRSX, MXD3 |
| (640, Inf] | FW340055, ANXA3, RNF24, STX3, CXCR1, PPP1R3B, LIMK2, AK127443, MXD1, CLEC4E, CXCR2, FCGR3B, ALPK1, GK, AK026714, NRBF2, KCNJ2, PHF21A, GNG10, DKFZp451J181, PELI1, ARL11, IL1R1, MSL1, LRRK2, RALB, FCGR2A, TLR1, SLC9A8, DENND5A, FNDC3B, CSF2RB, IL1RAP, FPR2, MNDA, MOSPD2, CEP63, TLR4, P2RY13, RNF149, NAMPT, B4GALT5, FPR1, CLEC7A, CTBS, BC028224 |

1. **Plot of some DE results**

Figure H: Expression of 12 of the 51 DE genes from the steelblue module in the pathways identified.


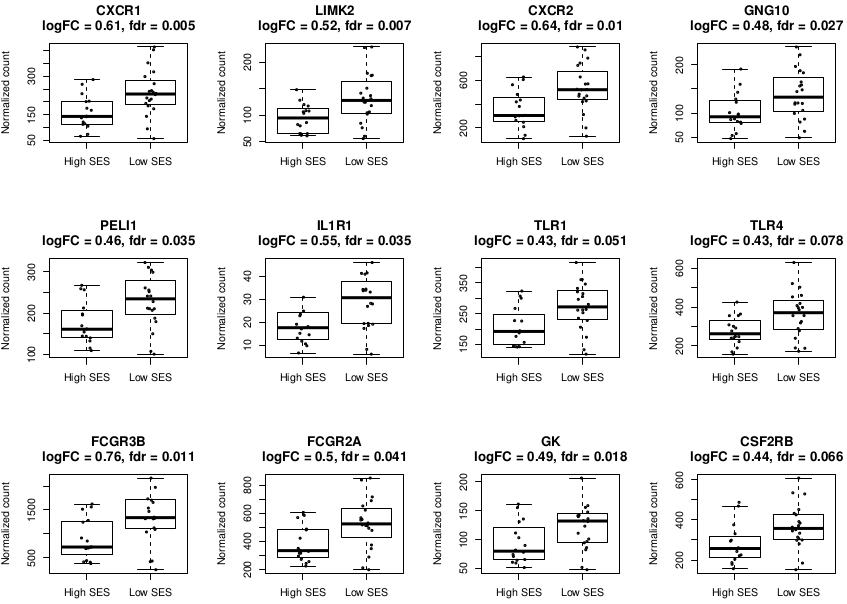


Figure I: Plots of expression to check CTRA DE genes reported as outliers; one sample with outlying expression on the low SES mainly drives the difference between the 2 groups.


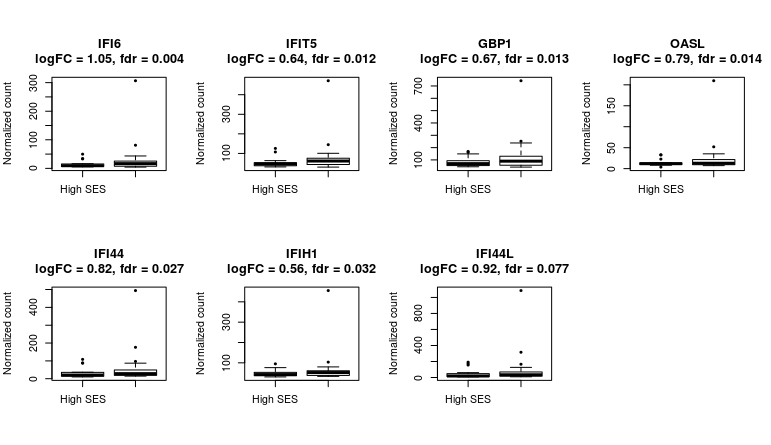


1. **References**

- Breiman, L., 2001. Random forests. Mach Learn 45, 5-32.
- Dewey, F.E., Perez, M.V., Wheeler, M.T., Watt, C., Spin, J., Langfelder, P., Horvath, S., Hannenhalli, S., Cappola, T.P., Ashley, E.A., 2011. Gene coexpression network topology of cardiac development, hypertrophy, and failure. Circ Cardiovasc Genet 4, 26-35.
- Genuer, R., Poggi, J.M., Tuleau-Malot, C., 2010. Variable selection using random forests. Pattern Recogn Lett 31, 2225-2236.
- Genuer, R., Poggi, J.M., Tuleau-Malot, C., 2015. VSURF: An R Package for Variable Selection Using Random Forests. R J 7, 19-33.
- Michener, C.D., R., S.R., 1957. A quantitative approach to a problem of classification. . Evolution 11, 490–499.
- Yip, A.M., Horvath, S., 2007. Gene network interconnectedness and the generalized topological overlap measure. Bmc Bioinformatics 8, 22.
